# Spatial Properties of Mismatch Negativity in Patients with Disorders of Consciousness

**DOI:** 10.1007/s12264-018-0260-4

**Published:** 2018-07-20

**Authors:** Xiaoyu Wang, Rao Fu, Xiaoyu Xia, Xueling Chen, Han Wu, Nicole Landi, Ken Pugh, Jianghong He, Fengyu Cong

**Affiliations:** 1 School of Biomedical Engineering, Faculty of Electronic Information and Electrical Engineering, Dalian University of Technology, Dalian, 116024 China; 2 Department of Neurosurgery, People’s Liberation Army General Hospital, Beijing, 100700 China; 3 Faculty of Linguistic Science, Beijing Language and Culture University, Beijing, 100083 China; 4 Haskins Laboratory, Yale University, New Haven, CT 06511 USA; 5 Department of Psychological Sciences, University of Connecticut, New Haven, CT 06269 USA; 6 Faculty of Information Technology, University of Jyvaskyla, Jyvaskyla, 40014 Finland

**Keywords:** EEG, Mismatch negativity, Disorder of consciousness, Vegetative state, Minimally conscious state

## Abstract

In recent decades, event-related potentials have been used for the clinical electrophysiological assessment of patients with disorders of consciousness (DOCs). In this paper, an oddball paradigm with two types of frequency-deviant stimulus (standard stimuli were pure tones of 1000 Hz; small deviant stimuli were pure tones of 1050 Hz; large deviant stimuli were pure tones of 1200 Hz) was applied to elicit mismatch negativity (MMN) in 30 patients with DOCs diagnosed using the JFK Coma Recovery Scale-Revised (CRS-R). The results showed that the peak amplitudes of MMN elicited by both large and small deviant stimuli were significantly different from baseline. In terms of the spatial properties of MMN, a significant interaction effect between conditions (small and large deviant stimuli) and electrode nodes was centered at the frontocentral area. Furthermore, correlation coefficients were calculated between MMN amplitudes and CRS-R scores for each electrode among all participants to generate topographic maps. Meanwhile, a significant negative correlation between the MMN amplitudes elicited by large deviant stimuli and the CRS-R scores was also found at the frontocentral area. In consequence, our results combine the above spatial properties of MMN in patients with DOCs, and provide a more precise location (frontocentral area) at which to evaluate the correlation between clinical electrophysiological assessment and the level of consciousness.

## Introduction

Recently, several electrophysiological studies have shown that event-related potentials (ERPs) can be applied to investigate auditory discrimination and its disorders in neurological patients [1]. In particular, mismatch negativity (MMN), a negative component of the ERP, has been used to assess the function of the auditory cortex in patients in a minimally conscious state (MCS) or in a vegetative state (VS) [2, 3]. MMN is a relatively automatic response to an occasional mismatched deviant stimulus that differs from repeated standard stimuli; it has a latency of 100 ms–250 ms at frontocentral and central scalp electrodes, and assesses whether the auditory system discriminates the different sounds [4, 5]. Compared with other ERP components, the advantage of MMN is that it can be recorded without attention, implying that it can also be recorded in unconscious patients.

The VS is a severe neurological syndrome caused by traumatic brain injury, hypoxia, or other etiologies [6, 7], in which behavioral signs of awareness are absent [8]. The MCS is described as a condition of minimal consciousness but with definite behavioral responses, and is distinct from VS [9]. The diagnostic criteria for VS and MCS are generally based on behaviors such as visual fixation and pursuit tracking. The CRS-R is usually used for the clinical assessment of patients with DOCs [10]. However, the diagnoses are extremely subjective, and have a low resolution in assessing consciousness [11]. MMN, as a functional brain investigation, should be used for accurate assessment of the level of consciousness (LOC) [12].

Several studies have used MMN to assess markers of cognition in VS or MCS. Fischer *et al*. applied an auditory stimulation paradigm, which consisted of standard tones, duration-deviant tones, and a novel stimulus to 27 patients with permanent VS or MCS, and found that some patients (mainly not due to anoxia) may be able to process sound deviance or novelty [3]. Boly *et al*. recorded MMN in 21 brain-damaged patients (8 VS and 13 MCS) and 22 healthy controls during a roving MMN paradigm, and found clearly different responses among controls and patients in MCS and VS [12]. Several statistical parametric maps of differential group responses over different time-windows were included to illustrate the significant interaction between the response amplitude and the LOC. Another study has shown that MMN increases with the recovery of consciousness, which implies that it can be used to predict the ability to recover from VS [13]. In the patient group, MMN was examined every 2 weeks during recovery from VS, while the normal group was only assessed once. And there was a negative correlation between LOC and MMN amplitude.

Although the latency of MMN is usually in the 100 ms–250 ms range, it may appear at a longer latency. Earlier studies that focused on the latency and amplitude of MMN showed that they are affected by the magnitude of the physical difference between the deviant and standard stimuli, and reflect the accuracy of perception [14]. Kotchoubey *et al*. compared the MMN between sine tone and complex tone stimuli in 79 patients with extremely severe brain injuries [2], and found that sine tones elicited an MMN of longer latency and smaller amplitude than complex tones. Risetti *et al*. recorded MMN in an auditory oddball paradigm in 11 patients diagnosed with VS and MCS [15]. All patients but one (due to anoxic etiology) showed MMN, and the MMN in VS patients occurred at a longer latency (339.2 ms ± 155.6 ms).

To date, in almost all studies of DOCs the participants were presented with only one type of deviant stimulus, which meant that analysis could only be performed between the responses to standard and deviant stimuli. However, the important differences in MMN properties between the responses to stimuli of different magnitudes of deviation were ignored (i.e. larger deviant stimuli elicit a larger MMN peak at a shorter latency) [16]. Furthermore, the paradigm with multiple types of deviant stimuli has been widely used on healthy participants [16–18] and has provided a comparison between different magnitudes of deviation [16]. Based upon this, in the present study we used two types of frequency-deviant stimuli to evaluate the MMN component as the outcome of the LOC. On the other hand, some studies have reported a correlation between behavioral index and MMN amplitude, but without topographic maps of the correlation coefficients as supporting evidence, and without revealing the spatial properties of MMN in experiments on patients with DOCs. In this study, we explored the correlation between MMN amplitude and clinical behavioral assessment for each electrode among all participants and revealed the spatial properties of the correlations between MMN and CRS-R scores in patients with DOCs using MMN as an index of the LOC.

## Methods

### Patients

Thirty patients were consecutively recruited at the PLA General Hospital. Patient #11 was excluded due to extreme CRS-R scores (Table 1, the score was not normally distributed), and patient #21 was excluded due to the extremely noisy electroencephalograph (EEG) data. Consequently, 28 patients with severe brain injury aged between 18 and 66 years (mean age ± SD, 44 ± 13) were enrolled. The time between the onset of coma and the evoked potential recording ranged between 1 and 12 months (mean 5 months).

**Table 1:** Detailed information about patients.

Patient	Gender (M,F)	Age (years)	Etiology	Months from event	CRS-R	Diagnosis
1	M	29	Cerebral hemorrhage	3	7	VS
2	F	40	Traumatic brain injury	11	7	VS
3	M	32	Cerebral hemorrhage	7	7	MCS–
4	M	40	Cardiac arrest	1.5	5	VS
5	M	40	Cardiac arrest	2.5	9	MCS–
6	M	55	Cardiac arrest	1.5	5	VS
7	M	33	Brainstem hemorrhage	1	8	MCS–
8	F	29	Cerebral hemorrhage	6	7	VS
9	F	53	Cerebral hemorrhage	2	16	MCS+
10	M	25	Cardiac arrest	6	12	MCS–
11	M	64	Aneurysm rupture	2	23	MCS+
12	F	36	Cardiac arrest	9	7	VS
13	F	60	Cerebral hemorrhage	3	7	VS
14	M	66	Traumatic brain injury	4	6	VS
15	M	65	Traumatic brain injury	4	6	VS
16	F	61	Cardiac arrest	2	6	VS
17	M	48	Cerebral hemorrhage	12	12	MCS+
18	F	48	Cerebral hemorrhage	4	10	MCS–
19	M	51	Brainstem hemorrhage	3	6	VS
20	F	60	Postoperative ramus myeloma	3.5	9	MCS–
21	M	53	Cardiac arrest	3	1	VS
22	M	38	Traumatic brain injury	3	7	VS
23	M	45	Bilateral vertebral artery occlusion	4	9	MCS–
24	M	18	Drowning	4	7	VS
25	M	29	Cardiac arrest	12	7	VS
26	F	53	Traumatic brain injury	1	3	VS
27	F	53	Traumatic brain injury	5	6	VS
28	F	30	Cardiac arrest	5	5	VS
29	M	61	Cerebral infarction	9	7	VS
30	M	42	Cerebral hemorrhage	3	9	MCS–

To determine the LOC of patients, we used the CRS-R scale to assess the behavioral scores [19]. The CRS-R scale is the most reliable and easiest to apply in routine use, comprising auditory, visual, motor, verbal, communication, and arousal functions, with a total score ranging between 0 (coma) and 23 (emergence from MCS). The patients were classified into 3 levels [20] (MCS+, MCS–, and VS; details in Table 1, as rated by clinicians).

Based on previous studies, MCS patients were subcategorized into MCS– (only showing non-reflex behavior such as visual pursuit, localization of noxious stimulation, and/or contingent behavior) and MCS+ (showing command following).

### Stimulus

**Fig. 1:**
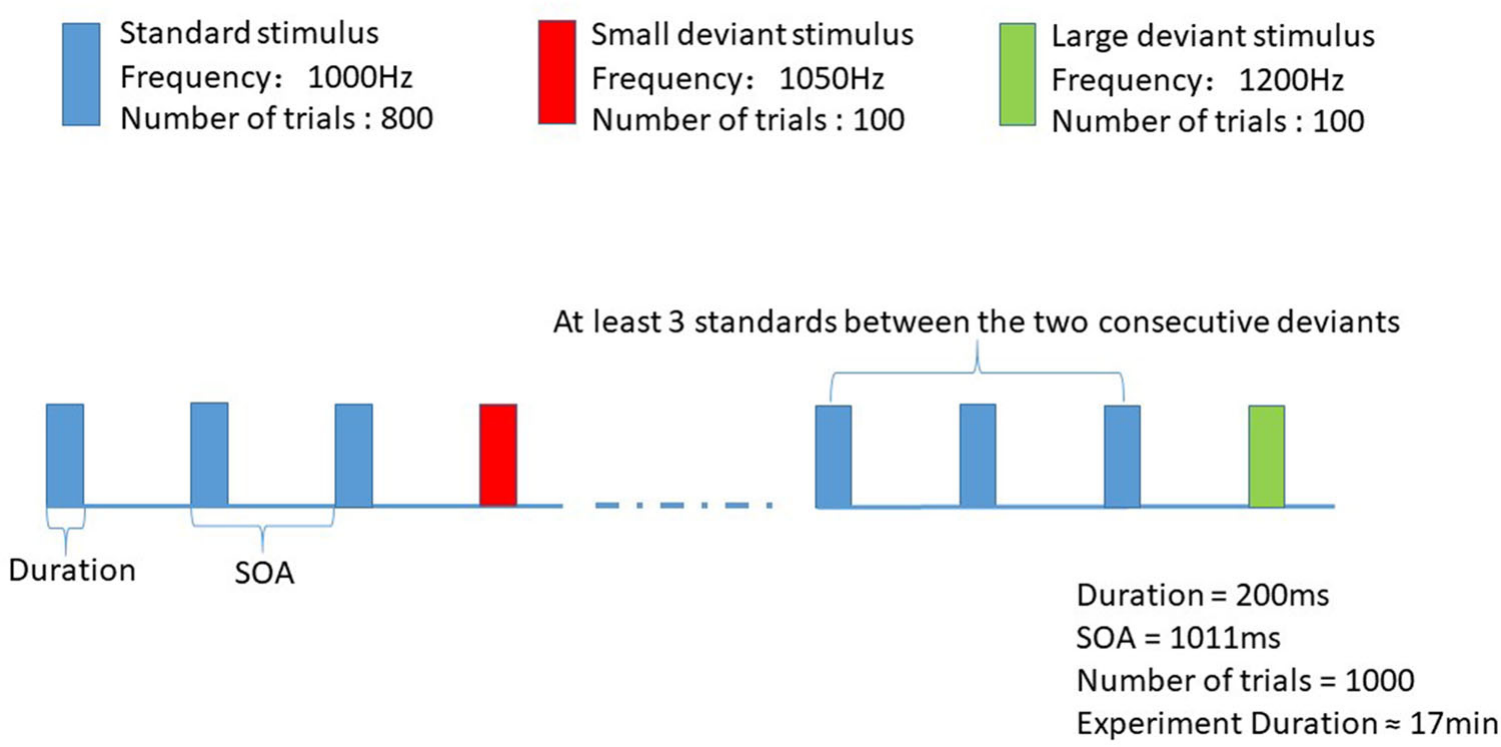
Stimulus sequences. 1000 pure sound stimuli (lasting for 200 ms) with SOA of 1011 ms were presented to a subject in order to elicit the MMN ERP response. The frequencies of standard, small deviant and large deviant stimuli were 1000 Hz, 1050 Hz and 1200 Hz, and the numbers of trials were 800, 100 and 100.

We used an oddball paradigm to elicit MMN; it consisted of one type of standard stimulus and two types of frequency-deviant stimulus. In each of the blocks, 1000 pure sound stimuli (lasting for 200 ms) with stimulus onset asynchrony of 1011 ms were presented to a patient in order to elicit the MMN ERP response. The standard sound stimuli (1000 Hz) were presented with a probability of 80%, that of the small deviant stimuli (1050 Hz) was 10%, and that of the large deviant stimuli (1200 Hz) was 10%. There were at least 3 standard stimuli between two consecutive deviants. Stimuli were uninterrupted and pseudo-randomly presented (Fig. 1). The whole experiment lasted for ~17 min. Stimulus sequences were programmed in the E-Prime 3.0 software (Psychology Software Tools, Pittsburgh, PA), and delivered through headphones.

### EEG Data Acquisition

Scalp EEGs were recorded at 21 electrodes (Fp1, Fp2, F3, F4, C3, C4, P3, P4, O1, O2, F7, F8, T3, T4, T5, T6, Fz, Cz, Pz, M1, and M2) according to the 10/20 International System using a Nicolet amplifier by Natus Neurology Inc. Data were sampled at 500 Hz with an online 0.1 Hz–100 Hz bandpass filter. Impedances were <10 KΩ and in most cases < 5 KΩ. Data were referenced online at the CPz electrode and re-referenced offline with the mean potential at the mastoids on both sides. Three channels were used to mark the onsets of the stimuli.

### Data Processing and Analysis

#### Preprocessing

EEG data were preprocessed with the EEGLAB toolbox [21]. The preprocessing consisted of rejecting artifacts in the time and frequency domains. Raw data were visually inspected by an experienced data analyzer to remove major artifacts caused by body movements. Channels with excessive artifacts were interpolated by the four nearby good-quality channels. Basic filters were applied in the following order: 50 Hz notch, 1 Hz high-pass, and 30 Hz low-pass filters.

#### Independent Component Analysis (ICA)

ICA is a well-known algorithm to remove electro-oculogram artifacts (blinks and eye movements). ICA was carried out on preprocessed data using the InfomaxICA algorithm [22]. The data from all 28 patients were spatially filtered by ICA to remove blink and eye movement artifacts.

### Extracting Epochs, Averaging, and Calculating the Difference Wave

Epochs were extracted after ICA. The EEG data were segmented into epochs of 700 ms, time-locked to stimulus onset, and included a pre-stimulus period of 100 ms (baseline). Then, the baseline was subtracted from each trial. Trials with an amplitude exceeding ± 75 μV were rejected. To balance the signal-to-noise ratio, only the standard stimuli before the deviant stimuli were averaged. In consequence, four sweeps were obtained, a small deviant sweep with the standard sweep before it, and a large deviant sweep with the standard sweep before it. The mean numbers of trials remaining for each stimulus were 90, 90, 90, and 89. After artifact rejection, the remaining trials were averaged according to the stimulus type for each patient. The difference wave is widely used to record MMN [18, 23]. To obtain a stable MMN, the deviant sweep minus the standard was used to calculate the difference wave.

### Wavelet Filtering

Wavelet filters are widely used to further improve the signal-to-noise ratio of MMN [24, 25]. Cong *et al*. provided a detailed introduction to wavelet filter design [26]. With regard to the number of levels for decomposition, the criterion was:

1 $$ {\text{L}} \approx \log_{2} {\text{Fs}} $$

where Fs is the sampling frequency, and L is the number of levels. Our sampling frequency was 500 Hz, and the number of levels for the wavelet decomposition was set to 9.

The roughly-defined bandwidth at a given level in wavelet decomposition is related to the sampling frequency and the corresponding frequency level [27] as:

2 $$ {\text{B}} = {\text{Fs}}/2^{{{\text{l}} + 1}} $$

where l = 1, …, L.

Table 2 shows rough estimates of the bandwidth at each level. The coefficients for D8, D7, D6, D5, and D4 were retained to construct the 1 Hz–30 Hz bandpass filter. We selected a reverse biorthogonal wavelet of the order of 6.8 to filter the difference wave [28].

**Table 2:** Division to frequency levels for wavelet decomposition.

Decomposition level	Decomposition label	Frequency range (Hz)	Bandwidth (Hz)
9	D9	0.48–0.94	0.48
8	D8	0.97–1.95	0.97
7	D7	1.95–3.90	1.95
6	D6	3.90–7.81	3.90
5	D5	7.81–15.62	7.81
4	D4	15.62–31.25	15.62
3	D3	31.25–62.5	31.25
2	D2	62.5–125	62.5
1	D1	125–250	125

### Statistical Analysis

MMN peak values and latencies were measured from the most negative peak occurring at 200 ms–300 ms after stimulus onset. Then, the mean amplitude within the time-window − 20 ms to 20 ms centered on the latency of the peak MMN component in the grand averaged waveform was taken as a feature for statistical analysis. Two-tailed *t*-tests were conducted to determine whether the MMN amplitudes with conditions (small and large stimuli) differed significantly from baseline and whether the MMN latency differed significantly among conditions. The mean amplitude within the fixed time-window among all 19 electrode nodes (the two reference electrodes M1 and M2 were excluded) was calculated as the MMN value. A repeated-measures analysis of variance (RMANOVA) was conducted with the conditions and electrode nodes as the factors to assess the differences in mismatch responses across conditions in each electrode. Follow-up *t*-tests were conducted to determine whether the conditions × electrodes interactions were significant, with the level of significance set at 0.05, and Greenhouse-Geisser corrections were applied where appropriate. Pearson’s linear correlation was used to calculate the correlation coefficients between MMN amplitudes at each electrode and CRS-R scores among all patients, with the level of significance set at 0.05; Bonferroni correction was applied.

## Results

### ERP Results

**Fig. 2:**
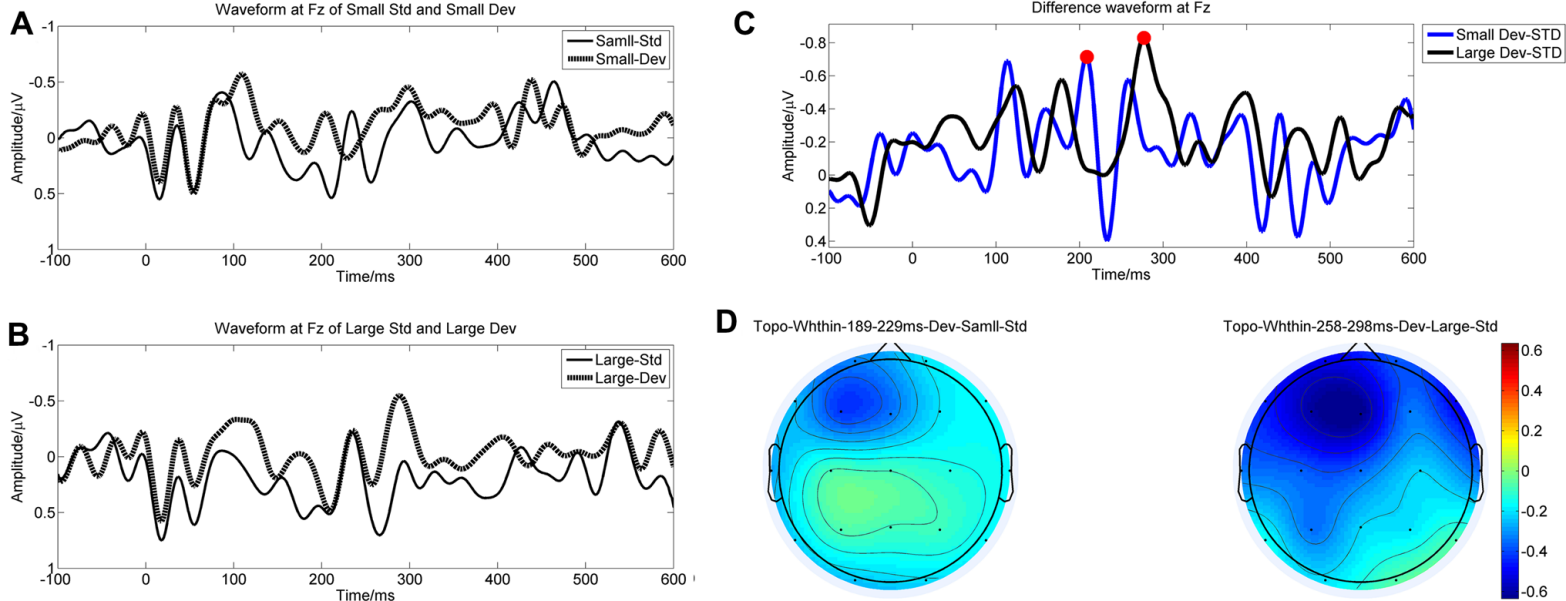
Grand averaged waveforms and topographies. **A** Grand averaged waveform of responses to small deviant stimuli and the sweep before it at electrode Fz. **B** Grand averaged waveform of responses to large deviant stimuli and the sweep before it at electrode Fz. **C** Grand averaged difference waves (at electrode Fz) of large (black line) and small (blue line) deviant stimuli. **D** Topography of mean MMN amplitude within the time window (time window of small deviant: 189 ms–229 ms; time window of large deviant: 258 ms–298 ms).

The grand averaged waveforms of the standard and deviant responses, along with the difference waves are shown in Fig. 2A–C. All the MMN amplitudes elicited by small and large deviant stimuli differed significantly from baseline (*t*_small_ = 5.208, *P*_small_ < 0.05; *t*_large_ = 5.996, *P*_large_ < 0.05).As noted in the data processing and analysis, the baseline was equal to zero after baseline correction. RMANOVA revealed a significant conditions × electrodes interaction (*F* = 2.525, *P* = 0.038). Follow-up *t*-test comparisons indicated that small deviant stimuli had significantly smaller mismatch responses at the frontocentral recording sites (Fp1, Fp2, Fz, F3, F4, F7, Cz, and C3; *t* > 2.071 for all, *P* < 0.05 for all), but no significant difference at the other recording sites (P3, P4, Pz, T3, T4, T5, T6, O1, O2, F8, and C4; *t* < 1.756 for all, *P* > 0.05 for all) (Table 3). In terms of latency, there was no significant difference between the different magnitudes of deviation (*t* = – 1.074, *P* = 0.292), and the grand averaged latencies to the small and large deviant stimuli were 239.61 ± 36.90 ms and 251.58 ± 38.36 ms. In terms of spatial distribution, the MMN component was mainly centered at the frontocentral area (Fig. 2D).

**Table 3:** Statistical parameters of the spatial properties of MMN.

Channel name	*t* value	rho of large deviant	rho of small deviant
Fp1	3.00*	0.15*	0.03
Fp2	2.39*	0.24*	0.06
F3	2.21*	0.13	0.06
F4	3.50*	0.11	0.03
C3	2.27*	0.08	0.08
C4	1.38	0.09	0.03
P3	0.80	0.03	0.07
P4	1.00	0.08	0.07
O1	−0.44	0.10	0.11
O2	0.54	0.13	0.14
F7	2.07*	0.05	0.01
F8	1.76	0.10	0.01
T3	0.49	0.02	0.07
T4	0.50	0.00	0.00
T5	−0.54	0.00	0.01
T6	−0.65	0.01	0.03
Fz	2.08*	0.14*	0.04
Cz	2.17*	0.24*	0.06
Pz	0.93	0.10	0.09

First column, electrodes; second column, *t*-test results of small and large deviant stimuli at each electrode; third and fourth column, correlation coefficients between MMN amplitudes induced by large and small deviant stimuli and CRS-R scores (*P < 0.05).

### Clinical and ERP Correlations

**Fig. 3:**
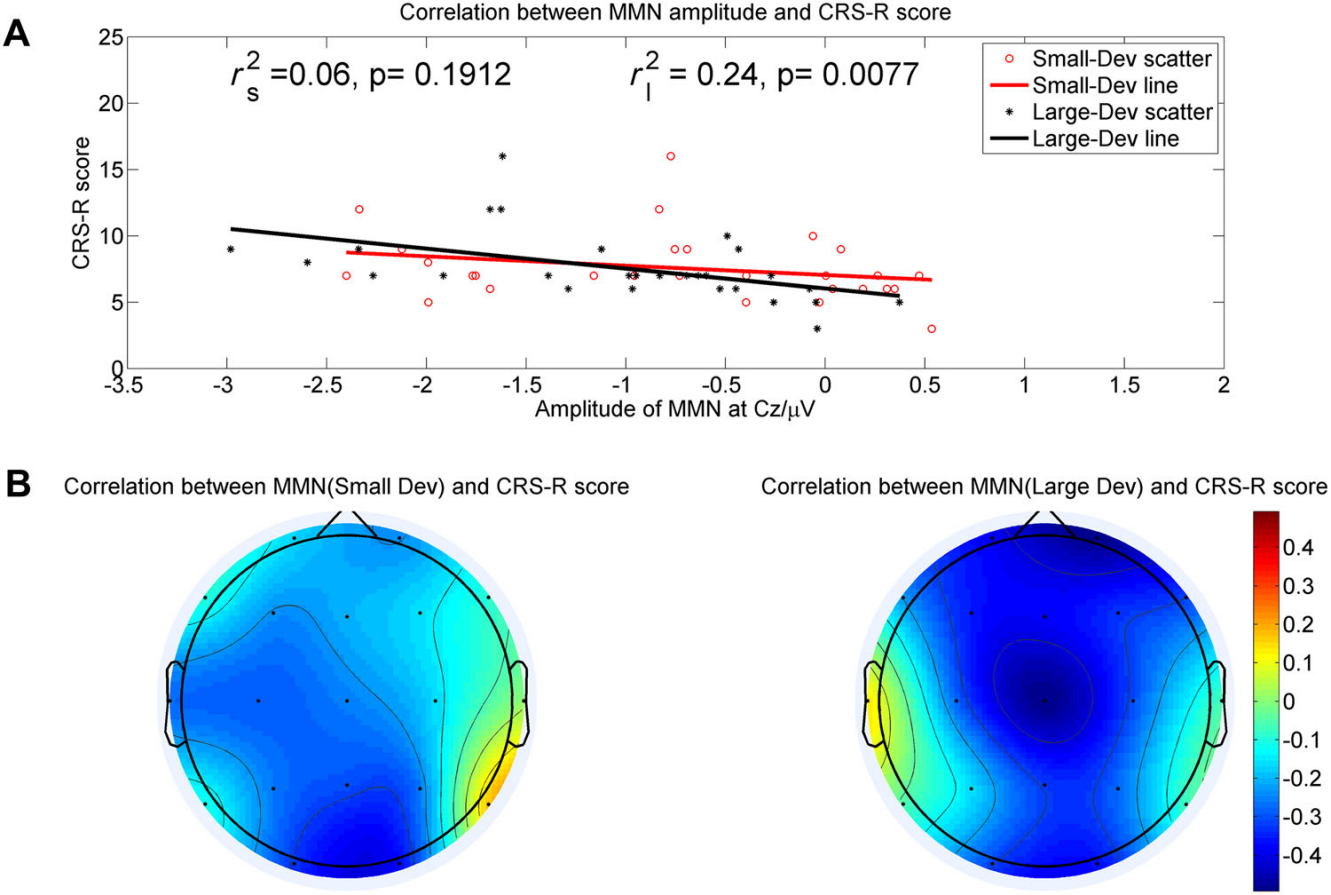
The correlation between MMN amplitude and CRS-R scores. **A** The correlation between MMN amplitude at electrode Cz and CRS-R scores (*r*_l2_/*r*_s2_ represents the correlation coefficient between MMN amplitude derived by large/small deviant stimuli and CRS-R scores). **B** The different shades on the topography represent the degree of association between the MMN amplitude across different electrode sites and CRS-R scores.

The total CRS-R scores were obtained before each ERP recording. The topographic maps of the correlation coefficients between MMN amplitudes and CRS-R scores are shown in Fig. 3B. A significantly negative correlation between CRS-R scores and MMN amplitudes elicited by large deviant stimuli was also found in the frontocentral area (Fp1, Fp2, Fz, and Cz; *P* < 0.05 for all, Table 3). Accordingly, as the CRS-R score increased, the absolute value of MMN amplitude became larger. The most significantly negative correlation was located at electrode Cz (*r*_s_^2^ = 0.06, *P* = 0.08; *r*_l_^2^ = 0.24, *P* = 0.007; *r*_s_^2^ and *r*_l_^2^ are the correlation coefficients between peak amplitudes of MMN components elicited by small and large deviant stimuli and CRS-R scores at electrode Cz, see Fig. 3A). However, such a significant correlation was not found between CRS-R scores and MMN amplitudes elicited by small deviant stimuli.

## Discussion

Here, we investigated the correlations between ERP components elicited by an oddball auditory paradigm in patients diagnosed with DOCs and the outcome of the clinical behavioral assessment, using every electrode to determine the spatial properties of such correlations. In the experiment, we adopted a two frequency-deviant oddball paradigm to elicit MMN components, and used the CRS-R score as the outcome of the LOC. The main findings can be summarized as follows: (1) there was a significant interaction effect between conditions (small and large deviant stimuli) and electrode nodes centered at the frontocentral area; (2) topographic maps of correlation coefficients between MMN amplitudes and CRS-R scores were generated; and (3) a significantly negative correlation between the MMN amplitude elicited by large deviant stimuli and the CRS-R scores was also found at the frontocentral area.

ERPs are routinely used as the outcome of the clinical electrophysiological assessment of patients with DOCs [3, 29, 30]. The first step to evaluate the feasibility of using MMN as the clinical outcome of LOC was to determine whether the MMN component was successfully derived. In terms of amplitude, there were significant differences between the MMN amplitudes elicited by both small and large deviant stimuli and baseline, implying that both the small and large deviant stimuli successfully elicited the MMN component in patients with DOCs.

**Fig. 4:**
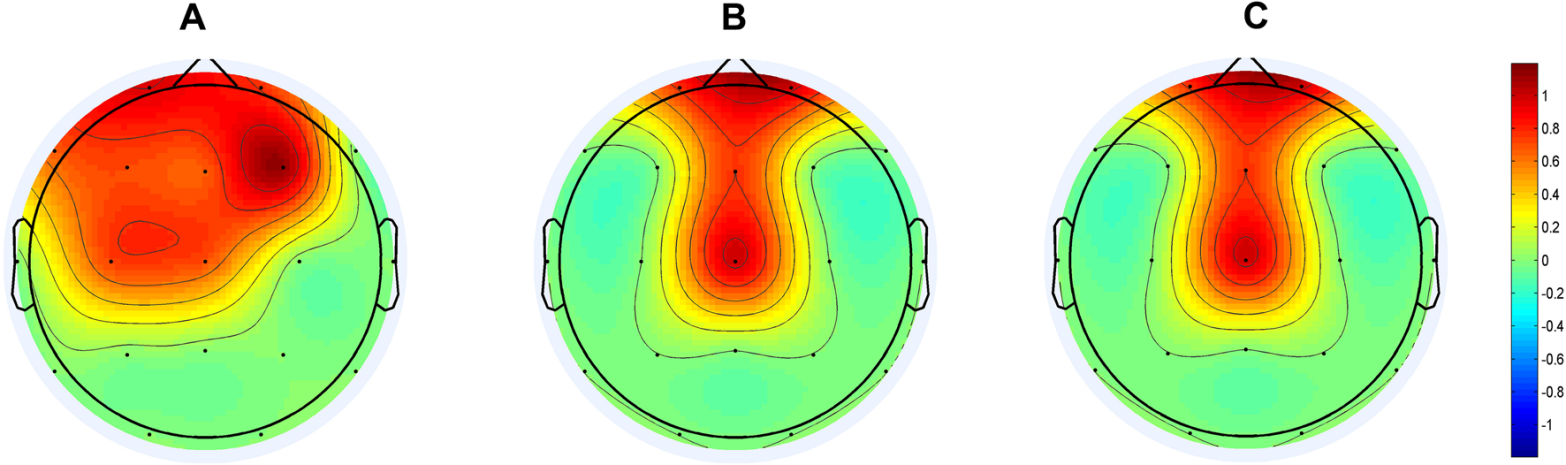
Spatial properties of the correlation between MMN and the CRS-R scores. **A** Statistical parametric maps showing scalp regions where there is a significant interaction effect between conditions (small and large deviant stimuli) and electrode nodes, with the threshold at *P* < 0.05. **B** Statistical parametric maps showing scalp regions where there is a significantly negative correlation between the MMN amplitudes elicited by large deviant stimuli and the CRS-R scores, with the threshold at *P* < 0.05. **C** Topography showing scalp regions where both the interaction effect (between conditions and electrode nodes) and negative correlation (between the MMN amplitudes elicited by large deviant stimuli and the CRS-R scores) are significant.

Previous studies have demonstrated an important MMN property in healthy individuals—there is a significant interaction between conditions (different magnitude of deviation) at electrode Fz and the magnitude of deviation [16]. We adopted a two frequency-deviant oddball paradigm and found a significant interaction effect between conditions (small and large deviant stimuli) and electrode nodes in patients with DOCs, especially in the frontocentral area (not only at electrode Fz). As a result, we concluded that the first spatial property of MMN was a significant interaction effect between conditions and electrode nodes, specifically at frontocentral recording sites (Fz, Cz, F3, and F4, Fig. 4A). In practice, there is a great need to apply multi-deviant oddball paradigms to enrich the method of analyzing MMN data from patients with DOCs. However, it is difficult to evaluate whether an MMN paradigm with multiple deviant stimuli works. Conversely, the first spatial property potentially provides a feasible method to solve the above difficulty. Namely, a feasible paradigm with multiple deviant stimuli should meet both rules that the MMN component is successful derived and the significant interaction effect between conditions and electrode nodes specifically appears at frontocentral recording sites.

With the aim of using the MMN component to assess the LOC, the correlation between MMN amplitudes and behavioral assessment plays a fairly important role. Based on the results of the correlations between the peak amplitudes of MMN and CRS-R scores at each electrode among all patients, the topographic maps of the correlation coefficients between MMN amplitudes and CRS-R scores suggested that such correlations are centered at distinct areas (Fig. 3B). Based on the results of statistical analysis, we concluded that the second spatial property of MMN was that the significantly negative correlation between the MMN amplitudes elicited by large deviant stimuli and the CRS-R scores was also located at the frontocentral area (Fig. 4B). From the above analysis, it was clear that not all electrodes had an interaction effect significantly correlated with the CRS-R scores, which has not been reported previously. We believe that the precise spatial features of MMN elicited by multiple deviant stimuli paradigm should meet both of the above spatial properties, namely a distinct area with a significant interaction effect between conditions and electrode nodes, and a significantly negative correlation between the MMN amplitudes and the CRS-R scores. As a consequence, we combined these two spatial properties and found a distinct area of correlation between MMN and the CRS-R scores in patients with DOCs centered at the frontocentral area (Fp1, Fp2, Fz, and Cz; Fig. 4C), which is a more precise location for evaluating the correlation between clinical electrophysiological assessment and LOC. Given such spatial properties, two further conclusions can be drawn: (1) they provide an efficient spatial indicator to monitor the effectiveness of training programs and predict the LOC; and (2) given that it is always difficult to record EEG signals with a large number of electrodes in patients, our study revealed that fewer electrodes can be used to measure MMN, which greatly improves efficiency.

However, the results of small deviant stimuli partly did not support our hypotheses. First, the latency of the small deviant tended to be shorter than the large deviant. As shown in Fig. 3, the grand averaged waveforms fluctuated in patients with DOCs and the peak amplitudes were almost the same within a time-window, which made it difficult to accurately identify the MMN component. Furthermore, the latencies and peak values of MMN among patients differed considerably, which may have resulted in a difference in latency. Second, the mismatch responses elicited by small deviant stimuli showed no significant correlation with CRS-R scores. Compared with the large deviant stimuli, these results implied that the frequency difference between standard and deviant stimuli is a key parameter for assessing the LOC of patients with DOCs. In our study, the small deviant stimuli (50 Hz difference in frequency) was able to elicit a minor MMN component, but could not be used to evaluate the LOC, but this can be adjusted in future. Another minor limitation of our study was that gender and age were not meticulously classified. With additional data, more stable and reliable results could be acquired.
